# Synthesis, chiroptical properties, and self-assembled nanoparticles of chiral conjugated polymers based on optically stable helical aromatic esters†

**DOI:** 10.1039/c7ra12652b

**Published:** 2018-01-03

**Authors:** Chao Zhang, Meng Li, Hai-Yan Lu, Chuan-Feng Chen

**Affiliations:** University of Chinese Academy of Sciences Beijing 100049 China haiyanlu@ucas.ac.cn; Beijing National Laboratory for Molecular Sciences, CAS Key Laboratory of Molecular Recognition and Function, Institute of Chemistry, Chinese Academy of Sciences Beijing 100090 China cchen@iccas.ac.cn +86-10-62554449

## Abstract

By Suzuki coupling reaction, three pairs of chiral conjugated polymers with optically stable helical aromatic ester subunits as the main-chain were designed and synthesized. Polymers (+)-P-P1 and (−)-M-P1, (+)-P-P2 and (−)-M-P2 showed strong fluorescence emission, strong mirror image CD and circularly polarized luminescence (CPL) signals in THF. For polymers (+)-P-P3 and (−)-M-P3, containing the tetraphenylethene (TPE) moiety, they not only showed obvious aggregation induced enhancement emission (AIEE), but also exhibited mirror image CD signals and aggregation-induced enhancement CPL signals in THF–water mixtures. Moreover, (+)-P-P3 and (−)-M-P3 could also form chiral nanoparticles by solvent evaporation induced self-assembly. Interestingly, it was further found that the size of the nanoparticles could be controlled by the changing of THF/water ratio, and their CPL properties were also shown.

## Abstract 2

Chiral conjugated polymers based on helical aromatic esters and self-assembled nanoparticles were prepared and showed strong fluorescence and CPL properties.

## Graphical abstract

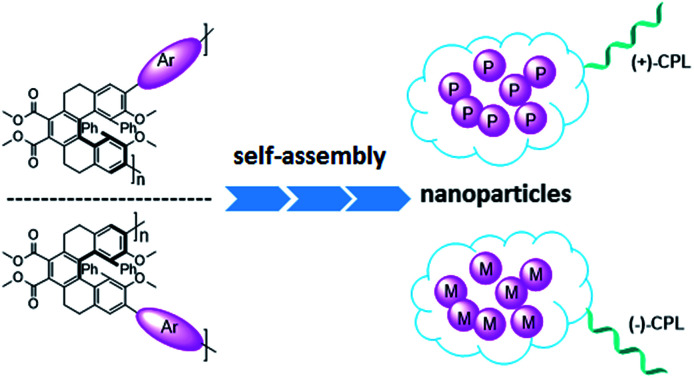

## Introduction

Conjugated polymers have drawn much attention during the last few decades for their wide potential application in organic photovoltaics (OPVs), organic light-emitting diodes (OLEDs), organic field-effect transistors (OFETs), bio-imaging, drug delivery and chemical sensors.^1^ Consequently, various achiral conjugated polymers including polypyrroles, polythiophenes, polyanilines and poly(*p*-phenylenevinylene)s have been developed so far.^2^ Chiral conjugated polymers are potentially useful in areas such as asymmetric synthesis, enantioselective sensing, and CPL materials.^3^ However, compared to the achiral ones, examples on chiral conjugated polymers are still very limited. Generally, chiral conjugated polymers could be formed by introducing chiral moieties into the conjugated polymer side chains or incorporating torsional, asymmetric, aromatic rings in the main chains.^4^ In 1997, Pu's group^5^ reported a kind of chiral conjugated polymer with a binaphthyl group as the main-chain. But such chiral polymers could be easily racemized and their fluorescence quenching in solid state often occurred. Thus, it is very important and meaningful to develop new kinds of optically stable conjugated polymers with specific structures and properties.

Helicenes^6^ and their derivatives have attracted increasing interest in recent years for their unique helical chirality, high fluorescence quantum efficiency, and CPL properties in both solution and solid state. So far, racemic helicene derivatives as the monomers have been used to synthesize the conjugated polymers.^7^ If an enantiopure helicene derivative with optical stability was used as the monomer, chiral conjugated polymers with specific chiroptical properties could thus be obtained. However, few such chiral conjugated polymers based on helicene derivatives were hitherto reported^8^ probably due to the easily racemization of simple helicenes or not easily available optical stable helicene derivatives.

Recently, we^9^ reported a kind of helical aromatic esters with strong blue emission and high dissymmetry factors. Especially, the easily available enantiopure 3,3′-dibromo-substituted helical aromatic esters with optical stability provide us an opportunity to fabricate new style of main-chain chiral conjugated polymers. In this paper, we report a new kind of chiral conjugated polymers. By introducing the chiral helical aromatic esters into the main-chain of conjugated polymers, we conveniently obtained three pairs of optically stable chiral conjugated polymers (+)-P-P1–3 and (−)-M-P1–3, which displayed mirror image CD signals and CPL properties. Especially, (+)-P-P3 and (−)-M-P3 containing tetraphenylethene (TPE) moiety showed obvious AIEE^10^ and aggregation-induced CPL in THF–water mixtures. Interestingly, it was also found that (+)-P-P3 and (−)-M-P3 could form chiral nanoparticles by solvent evaporation induced self-assembly, and size of the nanoparticle could be tuned by controlling of THF/water ratio.^11^ Moreover, the nanoparticles also showed CD and CPL-active. This represents the first example of self-assembled chiral nanoparticles based on chiral conjugated polymers with AIEE-active and CPL properties.^12^

## Results and discussion

### Synthesis and characterization

**Scheme 1:**
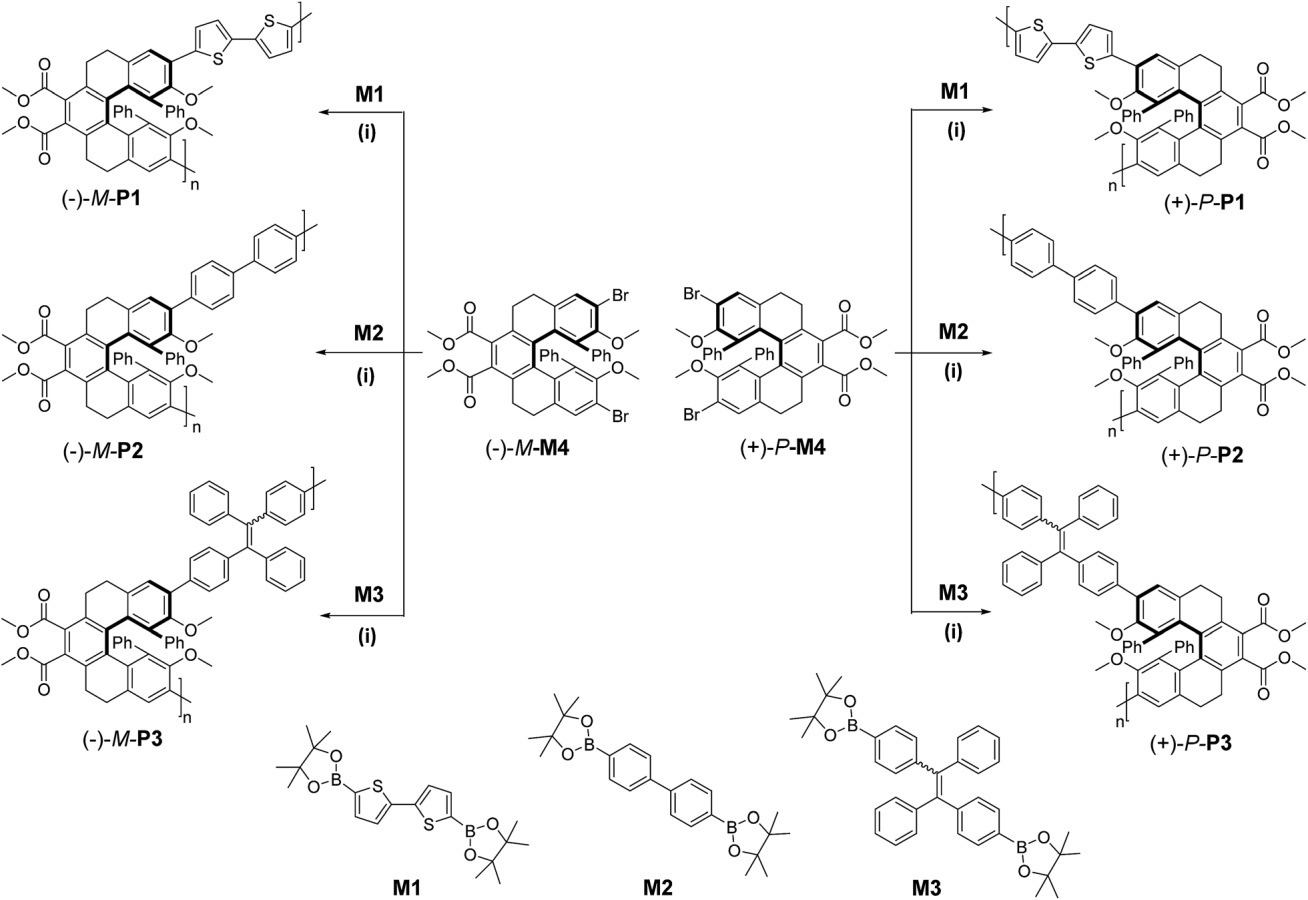
Synthesis of chiral conjugated polymers (+)-P-P1–3 and (−)-M-P1–3. M1: 2,2′-bithiophene-5,5′-diboronic acid bis(pinacol) ester; M2: 4,4′-biphenyldiboronic acid bis(pinacol) ester; M3: 1,2-diphenyl-1,2-bis(4-(4,4,5,5-tetramethyl-1,3,2-dioxaborolan-2-yl)phenyl)ethane; M4 was obtained by the literature's method.^9^ Conditions: (i) DMF (3 mL), toluene (2 mL), Pd(PPh_3_)_4_ (0.1 equiv.), K_2_CO_3_ (8 equiv.), 110 °C.

The synthetic procedures of the chiral conjugated polymers were depicted in Scheme 1, in which M1 and M2 were commercially available, while M3,^13^ (+)-P-M4 and (−)-M-M4 (ref. 9) were prepared according to the reported methods. Chiral conjugated polymers (+)-P-P1–3 and (−)-M-P1–3 could be synthesized in moderate to good yields (Table 1) by polymerization of the chiral monomers (+)-P-M4 and (−)-M-M4 with diboronic acid bis(pinacol) esters M1, M2, or M3*via* a Suzuki coupling reaction, respectively. All of the conjugated polymers were characterized by NMR and FTIR spectra. For example, in the ^1^H NMR spectra of (+)-P-P3 (Fig. S6†), the disappearing of the single peaks at 1.32 and 1.31 ppm for the methyl protons of M3 and existence of the wide characterized signals of the monomer implied the formation of the polymer. As shown in Table 1, the conjugated polymers all have comparable molecular weights, and they also showed good solubility in common solvents including CH_2_Cl_2_, CHCl_3_, CH_3_CN, and THF. According to the thermogravimetric analysis (TGA) (Fig. S14–16†), it was further found that the degradation temperatures (*T*_d_) of 5% weight loss of the polymers were all above 300 °C, indicating that the three pairs of conjugated polymers possessed the considerable high thermal stability, and this property might also make them have potential applications in optical materials.

**Table 1:** Molecular weights and yields of the polymers

Polymer	*M*_w_ a/kDa	*M*_n_ b/kDa	PDI^c^	Yield (%)
(+)-P-P1	11 989	8504	1.37	78
(−)-M-P1	10 666	8239	1.30	70
(+)-P-P2	15 054	12 777	1.28	71
(−)-M-P2	16 623	14 181	1.20	68
(+)-P-P3	11 333	10 353	1.11	61
(−)-M-P3	12 054	11 302	1.08	59

a *M*_w_: the weight-average molecular weight.

b *M*_n_: the number-average molecular weight.

c PDI: polydispersity indices (*M*_w_/*M*_n_).

### Photophysical properties of conjugated polymers in solution

**Fig. 1:**
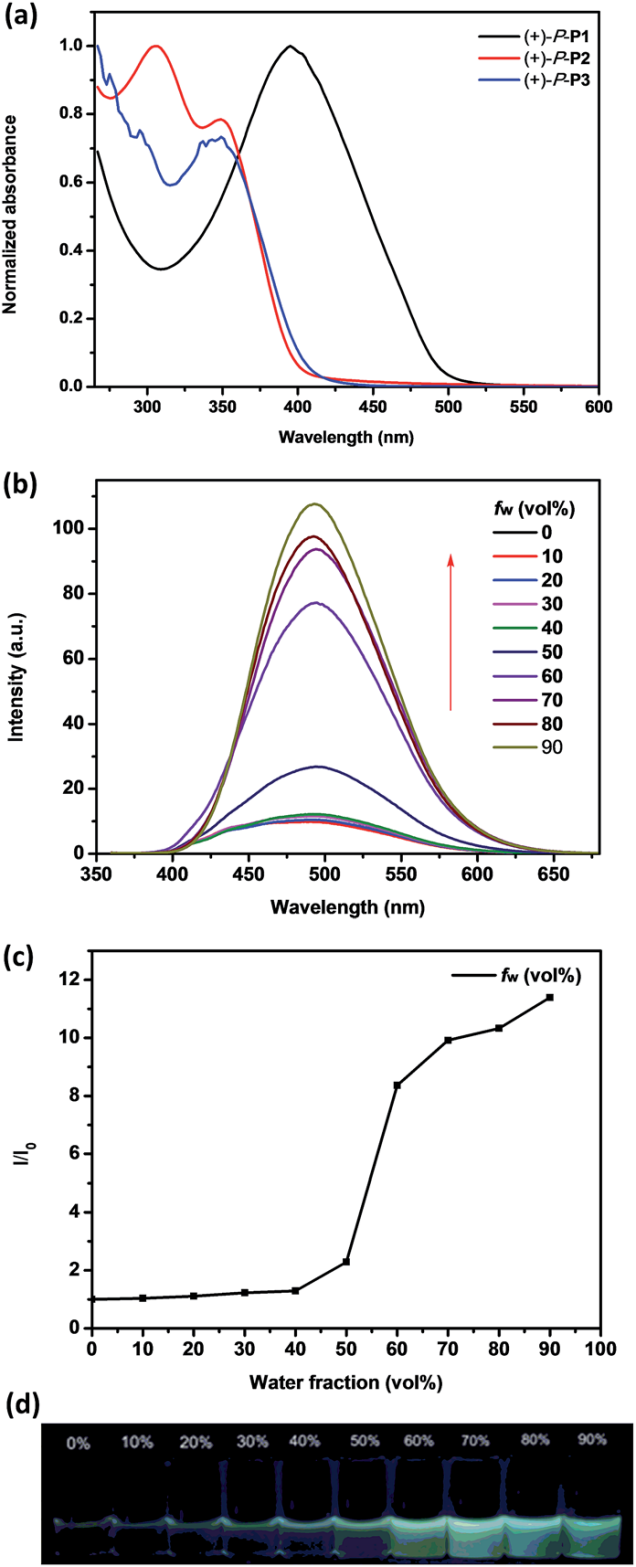
(a) UV-vis spectra of (+)-P-P1–3 in THF. (b) Fluorescence spectra of (+)-P-P3 in THF–water mixtures. (c) Plot of (*I*/*I*_0_) values of (+)-P-P3*versus* the compositions of the aqueous mixtures. (d) Images of (+)-P-P3 in THF–water under 365 nm UV lamp. *c* = 1.0 × 10^−4^ M corresponding to the helical aromatic esters moiety.

The absorption properties of chiral conjugated polymers (+)-P-P1–3 and (−)-M-P1–3 in THF were first investigated. As shown in Fig. 1a, the maximum absorption band of (+)-P-P1 was 395 nm, which was red shift compared with the monomer (+)-P-M4 (ref. 9) probably due to the electronic donor of the bithiophene. The absorption peak at 350 nm of (+)-P-P2 could be attributed to the helical aromatic esters skeleton, while the maximum absorption wavelength of polymer (+)-P-P2 at 305 nm might be attributed to the biphenyl group, which also implied the successful polymerization of (+)-P-M4 and M2. The maximum absorption of (+)-P-P3 was found to be less than 300 nm due to the characteristic absorption band of phenyl group, while an obvious absorption band at 350 nm corresponding to the n–π* transition of the C=O band of helical aromatic esters skeleton was also observed. The conjugated polymers (−)-M-P1–3 exhibited the similar optical properties, which were shown in Fig. S17.† The emission properties of the conjugated polymers in THF solution at room temperature were further investigated. As shown in Fig. S18,† the maximum emission bands of (+)-P-P1 and (−)-M-P1 in THF were all at 533 nm. Moreover, it was also found that the emission colour of (+)-P-P2 and (−)-M-P2 was near standard blue Commission International De L'Eclairage (CIE) coordinates of *x* = 0.14, *y* = 0.12 (Fig. S22†), which suggested that (+)-P-P2 and (−)-M-P2 might be new blue emission materials.

Since (+)-P-P3 and (−)-M-P3 contained the TPE moiety, we deduced that they should maintain the aggregation-induced emission^14^ properties. Consequently, we further investigated their fluorescence emission of the polymers in THF and THF–water mixtures at a fixed concentration of 1.0 × 10^−4^ M according to the TPE moiety. As shown in Fig. 1b, it was found that the maximum emission wavelength of (+)-P-P3 was at 500 nm, but with the increasing of the water fraction (*f*_w_) up to 90%, the emission intensity of (+)-P-P3 increased from 9.45 to 107.7, meanwhile the emission wavelength showed slight red shift. Especially, it was found that when the water fraction (*f*_w_) was changed from 50% to 60%, the emission intensity has a dramatical increase (Fig. 1c). This phenomenon indicated that the intramolecular rotation of the TPE moiety was restricted in this water fraction. The colour images obtained under a 365 nm UV lamp (Fig. 1d) also exhibited the gradually increased fluorescence intensity. These observations indicated that (+)-P-P3 possessed the obvious AIEE-active in THF–water mixtures. Under the same tested conditions, (−)-M-P3 showed the similar AIEE properties (Fig. S21†). However, the emission intensities of conjugated polymers (+)-P-P1 and (−)-M-P1, (+)-P-P2 and (−)-M-P2 decreased significantly with the increasing of the water fraction due to the aggregation-caused quenching (Fig. S19 and 20†), which are obviously different from those of polymers (+)-P-P3 and (−)-M-P3.

### Chiroptical properties

**Fig. 2:**
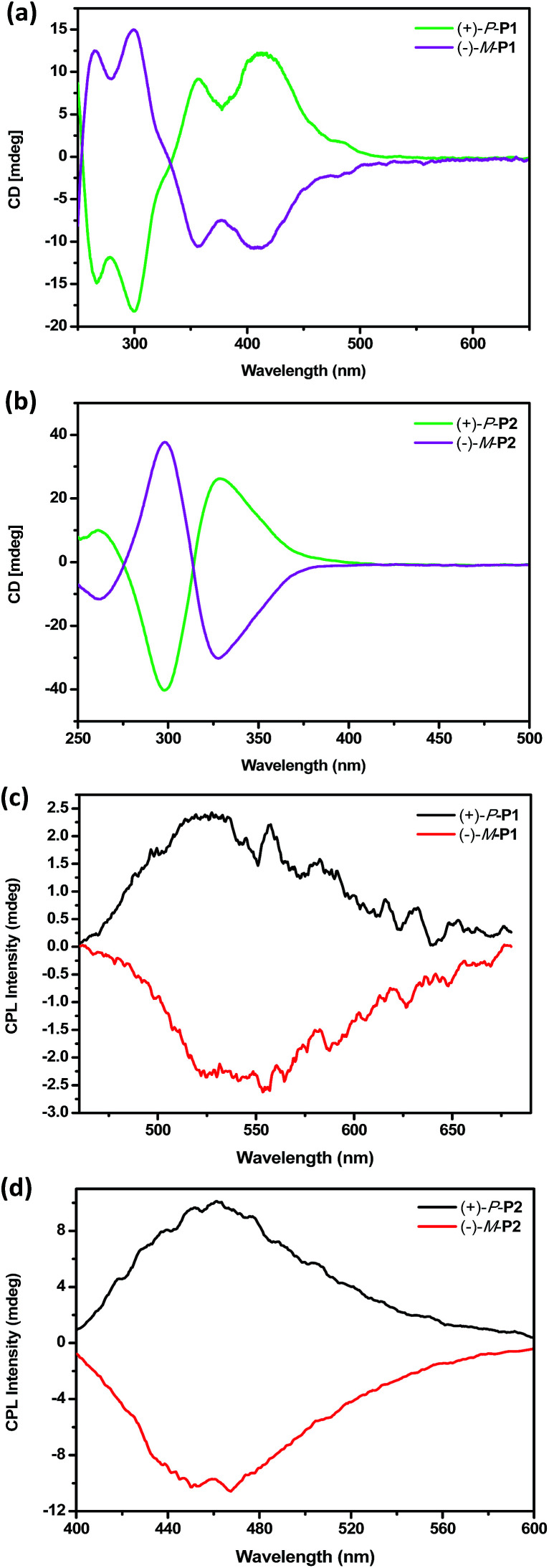
(a) CD spectra of (+)-P-P1 and (−)-M-P1 in THF; (b) CD spectra of (+)-P-P2 and (−)-M-P2 in THF; (c) CPL spectra of (+)-P-P1 and (−)-M-P1 in THF; (d) CPL spectra of (+)-P-P2 and (−)-M-P2 in THF. *c* = 1.0 × 10^−4^ M corresponding to the helical aromatic esters moiety.

**Fig. 3:**
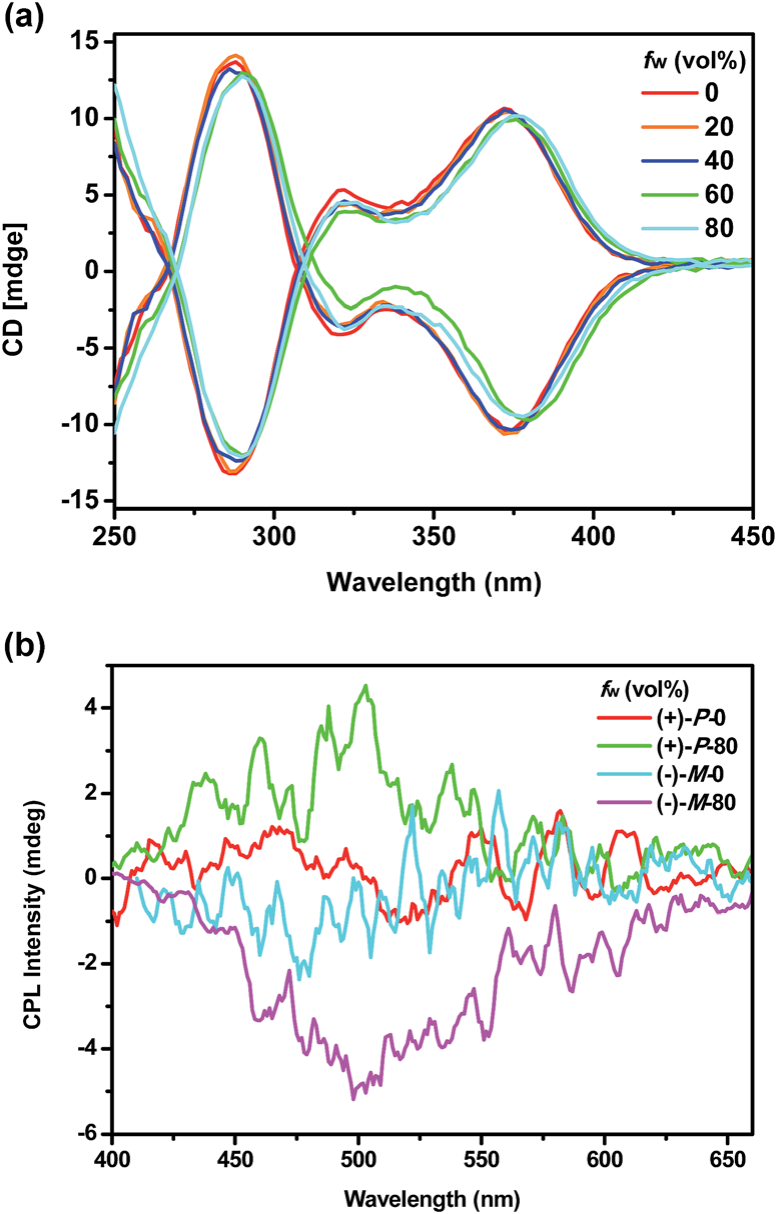
(a) CD spectra of (+)-P-P3 and (−)-M-P3 in THF–water mixtures; (b) CPL spectra of (+)-P-P3 and (−)-M-P3 in THF and THF–water mixtures (v/v, 20/80). *c* = 1.0 × 10^−4^ M corresponding to the TPE moiety.

To investigate the chiroptical properties of the chiral polymers, specific optical rotations of enantiomeric (+)-P-P1–3 and (−)-M-P1–3 were first measured and summarized in Table S1 (see ESI†). The results showed that the optical rotation values of (+)-P-P1–3 were all positive while the optical rotation values of (−)-M-P1–3 were negative. The CD spectra of (+)-P-P1–2 and (−)-M-P1–2 were further measured in THF at a same concentration of 1.0 × 10^−4^ M corresponding to the helical aromatic ester moiety. As shown in Fig. 2a and b, polymers (+)-P-P1 and (−)-M-P1, (+)-P-P2 and (−)-M-P2 showed mirror image CD signals in THF solution, respectively, and the *g*_abs_ values^15^ were found in the region of ±2.9 × 10^−4^ to ±6.5 × 10^−4^. Under the same tested conditions, (+)-P-P3 and (−)-M-P3 also showed the mirror-image CD profiles. (+)-P-P3 exhibited positive Cotton effect at about 375 nm and negative Cotton effect at about 285 nm, meanwhile (−)-M-P3 showed negative Cotton effect at about 375 nm and positive Cotton effect at about 285 nm. The *g*_abs_ values of (+)-P-P3 and (−)-M-P3 were 1.4 × 10^−4^ and −1.4 × 10^−4^, respectively, at about 375 nm. It was also found that almost no changes of the CD spectra were observed with the increase of water ratio (Fig. 3a), suggesting that the aggregation state showed almost no effect on the ground chirality of the polymers.

Since chiral conjugated polymers (+)-P-P1–3 and (−)-M-P1–3 all showed strong fluorescence, we then investigated their CPL properties. As shown in Fig. 2c and d, it was found that the polymers (+)-P-P1 and (−)-M-P1, and polymers (+)-P-P2 and (−)-M-P2 showed strong mirror-image CPL signals in THF solution, respectively. And the *g*_lum_ values were found in the region of ±3.1 × 10^−4^ to ±1.3 × 10^−3^. Under the same tested conditions, the (+)-P-P3 and (−)-M-P3 showed scarcely CPL signals in THF, which might be resulted from weak or no fluorescence of the polymers in solution due to the non-radiative energy conversion through intermolecular interactions. But when water was injected into the THF solution of (+)-P-P3 and (−)-M-P3, the CPL signals showed an obvious enhancement with the increase of the water fraction (Fig. 3b), which indicated that polymers (+)-P-P3 and (−)-M-P3 exhibited the aggregation-induced CPL active. The *g*_lum_ values^15^ of (+)-P-P3 and (−)-M-P3 were found to be about 8.3 × 10^−4^ and −8.3 × 10^−4^, respectively, which were in the reasonable range of most chiral conjugated polymers from 10^−5^ to 10^−2^.^16^

### Nanoparticles self-assembled by polymers (+)-P-P3/(−)-M-P3

**Fig. 4:**
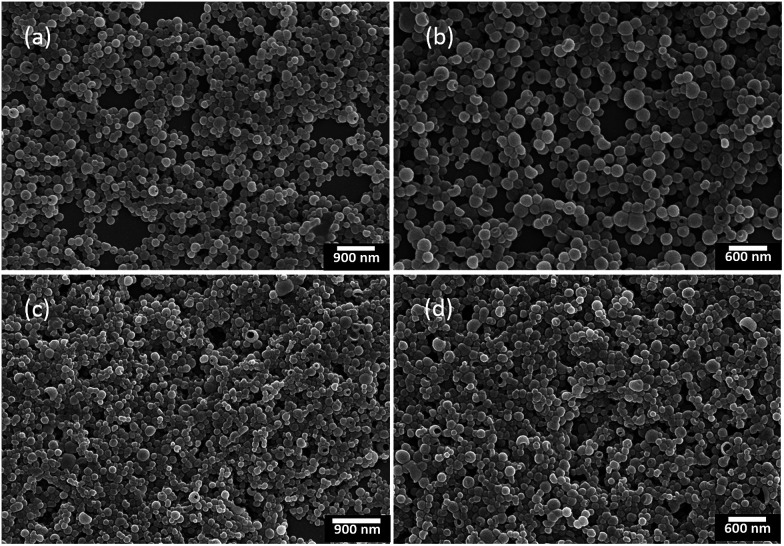
SEM images of the nanoparticles self-assembled by (a) (+)-P-P3 and (b) (−)-M-P3 from THF–water (v/v, 40/60); (c) (+)-P-P3 and (d) (−)-M-P3 from THF–water (v/v, 20/80).

Although the nanoparticles dispersed in aqueous solution could be widely used in bio-imaging, drug delivery and chemotherapy,^17^ the nanoparticles in solid state were also important for their potential applications in organic photovoltaics, organic light-emitting diodes and chemo/biosensors.^18^ On the other hand, it was known that chiral conjugated polymers (+)-P-P3 and (−)-M-P3 containing the TPE moiety showed strong fluorescence in aggregation state, which inspired us to further investigate their self-assembly behaviour and chiroptical properties of the chiral assembles. Consequently, by solvent evaporation induced self-assembly,^19^ we first dissolved (+)-P-P3 in THF (*c* = 1.0 × 10^−3^ M), and then injected into ultrapure water with an appropriate ratio, which could form a THF–water (40/60, v/v) mixture of the polymer at the concentration of 1.0 × 10^−4^ M. The mixture was further transferred onto neat quartz plates, and with the evaporation of THF and water for 6 h at room temperature, the nanoparticles self-assembled by (+)-P-P3 were successfully obtained. By using the same method, the nanoparticles self-assembled by (−)-M-P3 could also be conveniently obtained. The morphology and sizes of the self-assembled nanoparticles were investigated by scanning electron microscopy (SEM). As shown in Fig. 4, all of the obtained nanoparticles displayed regular sphericity. Meaningfully, it was also found that with THF/water ratios changing from 40/60 to 20/80 (v/v), the size of the assembled nanoparticles could be obvious decreased from 200–300 nm (Fig. 4a and b) to 100–250 nm (Fig. 4c and d). Therefore, the size of the nanoparticles could be conveniently adjusted by simply regulating the ratios of THF/water in the preparation.

**Fig. 5:**
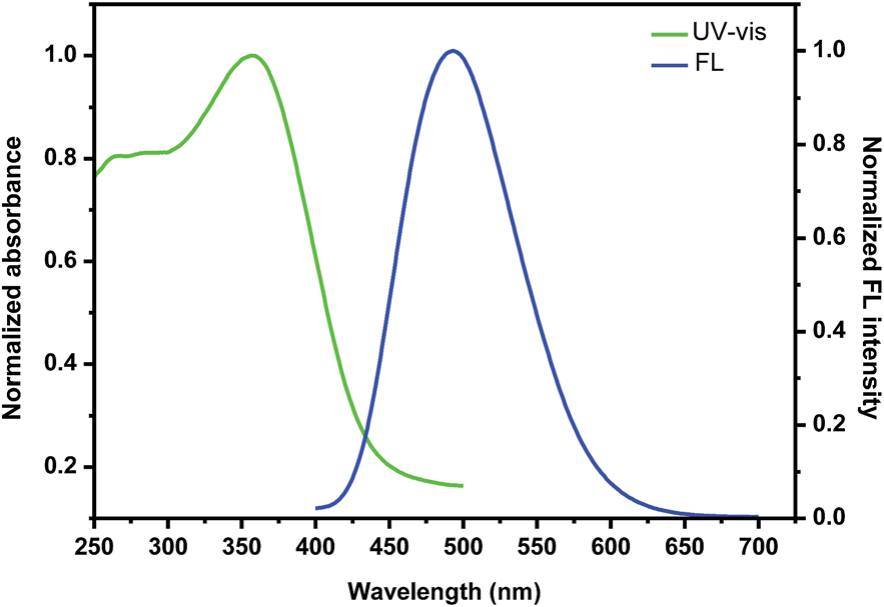
UV-vis spectra and fluorescence spectra of the nanoparticles assembled by (+)-P-P3.

With the nanoparticles in hand, we then investigated their photophysical properties. As shown in Fig. 5, the maximum absorption band of the nanoparticles self-assembled by (+)-P-P3 was found at 357 nm, which showed an slight red shift compared with that (350 nm) of the polymer in THF solution probably due to the stronger intermolecular interaction of the polymeric chains. It was also found that the nanoparticles showed intense emission at 502 nm, which were almostly consistent with that of the corresponding polymer in THF. Similarly, the nanoparticles self-assembled by (−)-M-P3 (Fig. S23†) displayed the intense absorption and emission, and the maximum wavelengths of both absorption and emission were consistent with those ones of the nanoparticles self-assembled by (+)-P-P3.

**Fig. 6:**
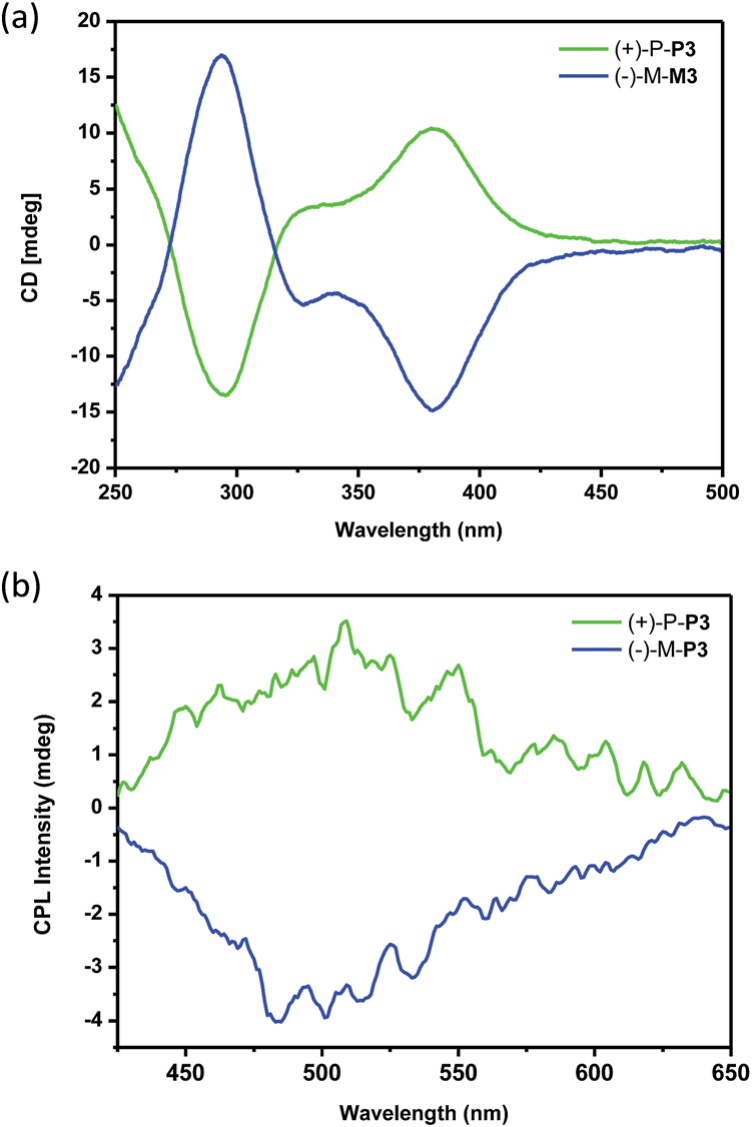
(a) CD spectra and (b) CPL spectra of the nanoparticles assembled by (+)-P-P3 and (−)-M-P3. The self-assembled nanoparticles were obtained from THF–water (20/80, v/v).

To investigate the chiroptical properties of the nanoparticles, their CD spectra were then measured. As shown in Fig. 6a, it was found that the nanoparticles self-assembled by (+)-P-P3 and (−)-M-P3 from THF/water (20/80, v/v) showed almost mirror-image CD signals, which were also similar to those of the polymers in THF solution. Moreover, the nanoparticles assembled by (+)-P-P3 and (−)-M-P3 exhibited strong Cotton effects at about 290 nm (negative signal for (+)-P-P3 and positive signal for (−)-M-P3) and about 375 nm (positive signal for (+)-P-P3 and negative signal for (−)-M-P3). The *g*_abs_ values of the nanoparticles from (+)-P-P3 and (−)-M-P3 were about 1.2 × 10^−3^ and −1.2 × 10^−3^ at about 375 nm, respectively, which implied that they had strong chirality in ground state. In order to investigate the excited chirality of nanoparticles of (+)-P-P3 and (−)-M-P3, we further investigated their CPL properties. As shown in Fig. 6b, it was found that the nanoparticles self-assembled by (+)-P-P3 displayed a positive CPL signal, and its *g*_lum_ value was about 0.95 × 10^−3^ at 502 nm. Meanwhile, the nanoparticles assembled by (−)-M-P3 displayed a negative CPL signal with *g*_lum_ value about −1.04 × 10^−3^ at 502 nm. The almost mirror-image CD and CPL signals of the nanoparticles self-assembled by (+)-P-P3 and (−)-M-P3 indicated the excellent chiroptical properties in aggregation state. We also tested the CPL properties of (+)-P-P3 and (−)-M-P3 in the film state (Fig. S24†). It was found that the *g*_lum_ values of (+)-P-P3 and (−)-M-P3 in film state were about ±0.88 × 10^−3^ at 502 nm, which were similar to the *g*_lum_ values of chiral nanoparticles assembled by (+)-P-P3 and (−)-M-P3.

## Conclusions

In summary, we have designed and synthesized three pairs of chiral conjugated polymers (+)-P-P1–3 and (−)-M-P1–3 by Suzuki coupling reactions between the optically stable 3,3-dibromo-substituted helical aromatic ester enantiomers and diboronic acid bis(pinacol) esters. It was found that chiral conjugated polymers (+)-P-P1 and (−)-M-P1, (+)-P-P2 and (−)-M-P2 showed strong fluorescence emission, mirror image CD signals and strong mirror image CPL signals in solution. For polymers (+)-P-P3 and (−)-M-P3 containing TPE moiety, they not only showed mirror image CD signals, but also exhibited obvious AIEE-active and aggregation-induced CPL properties in THF–water mixtures. Moreover, we also found that (+)-P-P3 and (−)-M-P3 could self-assemble into regular chiral nanoparticles, and the sizes could be turned by changing the THF/water ratio. Especially, it was found that the nanoparticles of (+)-P-P3 and (−)-M-P3 not only maintained the strong fluorescence emission of the polymers, but also showed strong mirror image CPL signals. To the best of our knowledge, the results presented herein represent the first examples of chiral conjugated polymers with AIEE-active and CPL properties based on the monomers with helical chirality, which might be potential applications in CPL optical materials.

## Experimental

### Materials and methods

All the solvents and reagents were commercially available. Nuclear magnetic resonance (NMR) spectra were obtained from a Bruker Avance 300 spectrometer and 500 spectrometer, and reported in parts per million (ppm) from the internal standard TMS. Fourier transform infrared spectrometer (FTIR) spectra were recorded on a Nicolet 6700 spectrometer. UV-vis spectra were obtained from PerkinElmer Lambda 950. Fluorescence spectra were performed by Hitachi F-7000. Circular dichroism were recorded on a JASCO J-810 spectropolarimeter. Circularly polarized luminescence (CPL) spectra were acquired on a JASCO CPL-200 spectropolarimeter. Thermogravimetric analyses (TGA) were obtained from a Netzsch STA449F3 instrument under N_2_ atmosphere. Molecular weight were measured by gel permeation chromatography (GPC) using a Waters 1515 HPLC pump, and THF was used as solvent relative to polystyrene standards. Scanning electron microscopy (SEM) images were obtained from SU8020, and transmission electron microscope (TEM) images were obtained from Hitachi 7700. Dynamic light scattering (DLS) using an ALV CGS-3.

### Synthetic procedures

#### Synthesis of (+)-P-P1 and (−)-M-P1

To a mixture of (+)-P-M4 (150 mg, 0.20 mmol), 2,2′-bithiophene-5,5′-diboronic acid bis(pinacol) ester (117 mg, 0.28 mmol), Pd(PPh_3_)_4_ (34 mg, 15% mmol) and K_2_CO_3_ (200 mg 1.45 mmol) in a 10 mL seal tube under N_2_ atmosphere were added 3 mL DMF and 2 mL toluene. The mixture was stirred in 110 °C for 72 h, and then cooled to room temperature. The reaction mixture was poured into 100 mL ethyl acetate, and then washed with 40 mL water for several times. Further purification was performed by redissolving the polymer in THF, precipitating again with cooled methanol, and then filtration. The polymer was dried in vacuum to afford (+)-P-P1 (182.2 mg, yield: 78%) as a blackish green solid. GPC: *M*_n_ = 8504, *M*_w_ = 11 989, PDI = 1.37. ^1^H NMR (300 MHz, CDCl_3_): ^1^H NMR (300 MHz, CDCl_3_): *δ* 7.51 (br, 2H), 7.15–6.85 (m, 12H), 6.25 (br, 2H), 3.91 (s, 6H), 2.85 (s, 6H), 2.75–2.65 (m, 2H), 2.50–2.35 (m, 2H), 2.30–2.20 (m, 2H), 1.48–1.40 (m, 2H). FTIR (KBr, cm^−1^): 3067, 3053, 3023, 2948, 2929, 2851, 1731, 1575, 1440, 1407, 1374, 1271, 1240, 1194, 1170, 1151, 1017, 801, 698. According to the same method, (−)-M-P1 could be obtained in 70% yield. *M*_n_ = 8239, *M*_w_ = 10 666, PDI = 1.30. ^1^H NMR (300 MHz, CDCl_3_): ^1^H NMR (300 MHz, CDCl_3_): *δ* 7.52 (br, 2H), 7.14–6.85 (m, 12H), 6.24 (br, 2H), 3.91 (s, 6H), 2.85 (s, 6H), 2.76–2.65 (m, 2H), 2.50–2.35 (m, 2H), 2.31–2.21 (m, 2H), 1.50–1.45 (m, 2H). FTIR (KBr, cm^−1^): 3070, 3053, 3023, 2947, 2926, 2848, 1731, 1574, 1440, 1374, 1271, 1211, 1194, 1150, 1017, 801, 744, 698.

#### Synthesis of (+)-P-P2 and (−)-M-P2

To a mixture of (+)-P-M4 (100 mg, 0.13 mmol), 4,4′-biphenyldiboronic acid bis(pinacol) ester (58 mg, 0.14 mmol), Pd(PPh_3_)_4_ (21.9 mg, 15% mmol) and K_2_CO_3_ (150 mg 1.09 mmol) in a 10 mL seal tube under N_2_ atmosphere were added 3 mL DMF and 2 mL toluene. The mixture was stirred in 110 °C for 96 h, and then cooled to room temperature. The reaction mixture was poured into 100 mL ethyl acetate, and then washed with 40 mL water for three times. Further purification was performed by re-dissolving the polymer in THF, precipitating again with cooled methanol, and then filtration. The polymer was dried in vacuum to afford (+)-P-P2 (109.0 mg, yield: 71%) as a white solid. GPC: *M*_n_ = 12 777, *M*_w_ = 15 054, PDI = 1.28. ^1^H NMR (300 MHz, CDCl_3_): *δ* 7.81 (br, 6H), 7.10–6.75 (m, 12H), 6.26 (br, 2H), 3.93 (br, 6H), 2.76 (br, 6H), 2.74–2.70 (m, 2H), 2.55–2.45 (m, 2H), 2.30–2.20 (m, 2H), 1.52–1.42 (m, 2H). FTIR (KBr, cm^−1^): 3078, 3056, 3025, 2950, 2927, 2853, 1731, 1589, 1469, 1456, 1437, 1375, 1273, 1262, 1200, 1171, 1148, 1097, 1039, 824, 699, 541. According to the same method, (−)-M-P2 could be obtained in 68% yield. GPC: *M*_n_ = 14 181, *M*_w_ = 16 623, PDI = 1.20. ^1^H NMR (300 MHz, CDCl_3_): *δ* 7.82 (br, 6H), 7.20–6.75 (m, 12H), 6.26 (br, 2H), 3.93 (br, 6H), 2.89 (br, 6H), 2.80–2.70 (m, 2H), 2.57–2.45 (m, 2H), 2.32–2.20 (m, 2H), 1.50–1.40 (m, 2H). FTIR (KBr, cm^−1^): 3081, 3051, 3026, 2950, 2927, 2856, 1733, 1577, 1501, 1458, 1439, 1375, 1273, 1202, 1171, 1094, 1031, 822, 699.

#### Synthesis of (+)-P-P3 and (−)-M-P3

To a mixture of (+)-P-M4 (150 mg, 0.20 mmol), 1,2-diphenyl-1,2-bis(4-(4,4,5,5-tetramethyl-1,3,2-dioxa-borolan-2-yl)phenyl)ethane (140 mg, 0.24 mmol), Pd(PPh_3_)_4_ (21.9 mg, 15% mmol) and K_2_CO_3_ (150 mg 1.09 mmol) in a 10 mL seal tube under N_2_ atmosphere were added 3 mL DMF and 2 mL toluene. The mixture was stirred in 110 °C for 7 d, and then cooled to room temperature. The mixture was poured into 100 mL ethyl acetate, and then washed with 40 mL water for three times. Further purification could be performed by redissolving the polymer in THF, precipitating again with cooled methanol, and then filtration. The polymer was dried in vacuum to afford (+)-P-P3 (162.6 mg, yield: 61%) as a green solid. GPC: *M*_n_ = 10 353, *M*_w_ = 11 333, PDI = 1.11. ^1^H NMR (500 MHz, CDCl_3_): *δ* 7.45–7.34 (m, 4H), 7.13–7.06 (m, 18H), 7.05–6.85 (m, 6H), 6.19 (br, 2H), 3.90 (br, 6H), 2.70–2.64 (m, 6H), 2.45–2.38 (m, 2H), 2.22–2.15 (m, 2H), 1.70–1.63 (m, 2H), 1.42–1.35 (m, 2H). FTIR (KBr, cm^−1^): 3081, 3053, 3026, 2948, 2928, 2851, 1734, 1599, 1457, 1437, 1303, 1274, 1200, 1172, 1148, 1098, 1038, 750, 698. According to the same method, (−)-M-P3 could be obtained in 59% yield. GPC: *M*_n_ = 11 302, *M*_w_ = 12 054, PDI = 1.08. ^1^H NMR (500 MHz, CDCl_3_): *δ* 7.46–7.36 (m, 4H), 7.13–7.05 (m, 18H), 7.05–6.80 (m, 6H), 6.19 (br, 2H), 3.91–3.80 (m, 6H), 2.70–2.60 (m, 6H), 2.45–2.38 (m, 2H), 2.22–2.15 (m, 2H), 1.70–1.63 (m, 2H), 1.41–1.35 (m, 2H). FTIR (KBr, cm^−1^): 3078, 3053, 3022, 2948, 2925, 2852, 1733, 1596, 1457, 1441, 1273, 1262, 1200, 1171, 1148, 1097, 1030, 801, 749, 698.

## Conflicts of interest

There are no conflicts to declare.

## Supplementary Material

RA-008-C7RA12652B-s001
