# Supplementary material for: Synthesis, chiroptical properties, and self-assembled nanoparticles of chiral conjugated polymers based on optically stable helical aromatic esters
Source: RSC Adv. 2018 Jan 3;8(2):1014–21. doi: 10.1039/c7ra12652b (PMC9076947; doi:10.1039/c7ra12652b)
Supplement: RA-008-C7RA12652B-s001 [file RA-008-C7RA12652B-s001.pdf]

Electronic Supplementary Material (ESI) for RSC Advances.
This journal is © The Royal Society of Chemistry 2017

Electronic Supplementary Information

Synthesis, chiroptical properties, and self-assembled nanoparticles of chiral conjugated polymers based on optically stable helical aromatic esters

Chao Zhang,[a] Meng Li,[b] Hai-Yan Lu,*[a] and Chuan-Feng Chen*[ab]

[a]*University of Chinese Academy of Sciences, Beijing 100049, China. E-mail: haiyanlu@ucas.ac.cn.*

[b]*Beijing National Laboratory for Molecular Sciences, CAS Key Laboratory of Molecular Recognition and Function, Institute of Chemistry, Chinese Academy of Sciences, Beijing 100190, China. E-mail: cchen@iccas.ac.cn.*

Contents

S1. Copies of ^{1}H NMR spectra

S2. Copies of FTIR spectra

S3. TGA thermogram of the polymers

S4. UV-vis and fluorescence spectra of the polymers

S5. Emission color coordinate of (+)-*P*-**P2 and** (-)-*M*-**P2**

S6. Optical rotation values of (+)-*P*-**P1-3 and** (-)-*M*-**P1-3**

S7. UV-vis spectra and fluorescence spectra of the nanoparticles assembled by (-)-*M*-**P3**

S8. CPL spectra of the (+)-*P*-**P3 and** (-)-*M*-**P3 in the film state**

S1. Copies of the ^{1}H NMR spectra

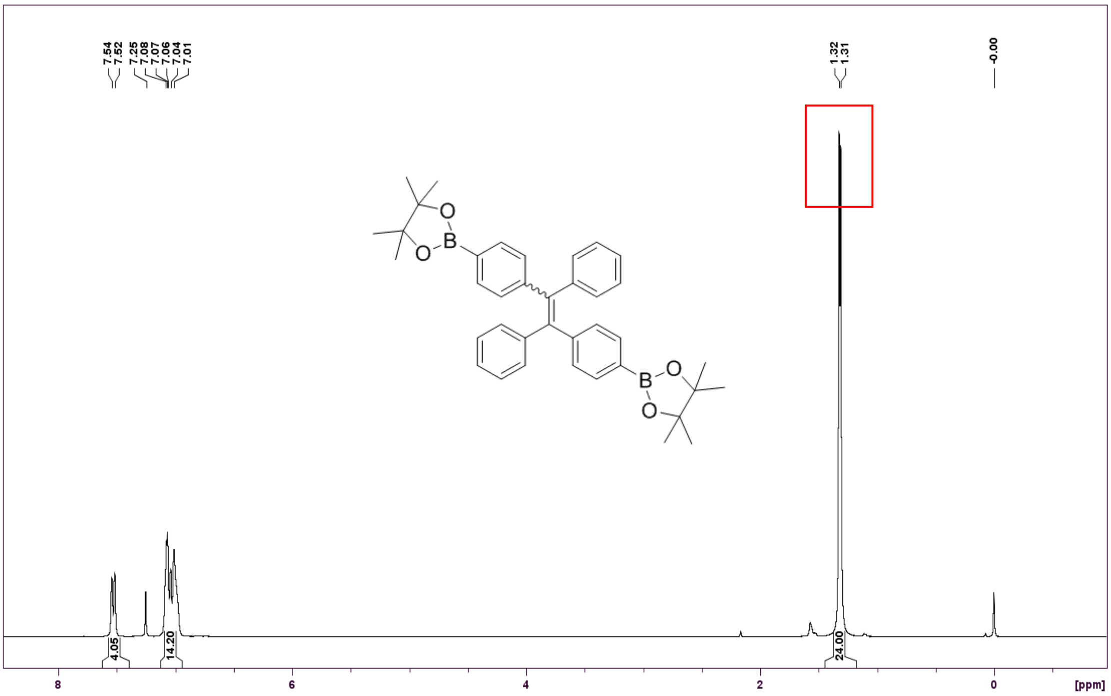

Fig. S1 ^{1}H NMR spectrum (300 MHz, $CDCl_3$) of **TPE**.[1]

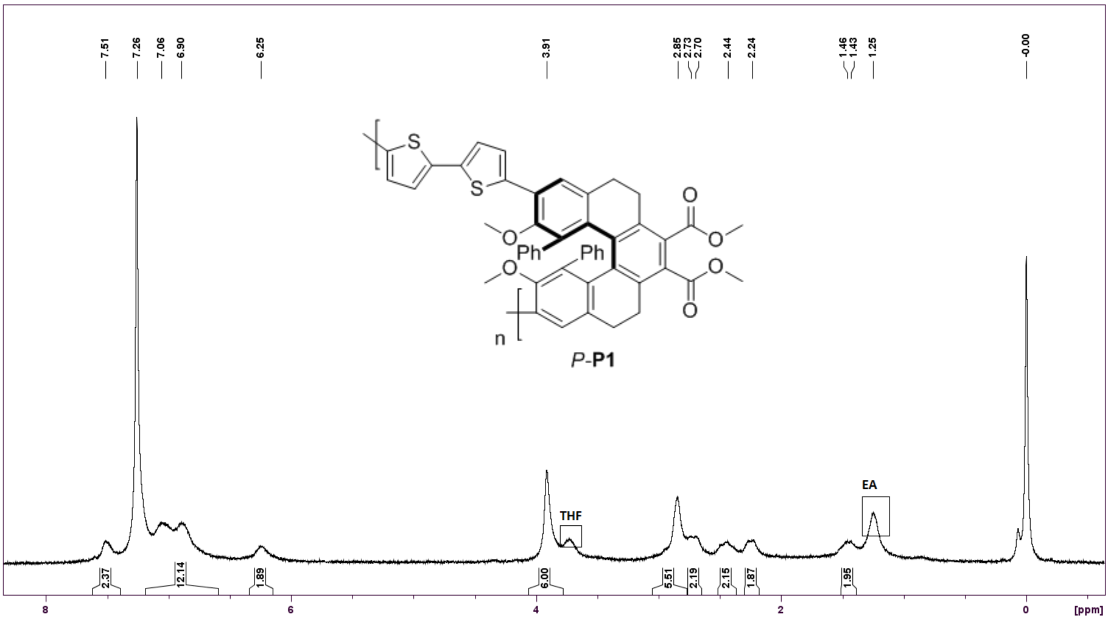

Fig. S2 ^{1}H NMR spectrum (300 MHz, $CDCl_3$) of (+)-*P*-**P1**.

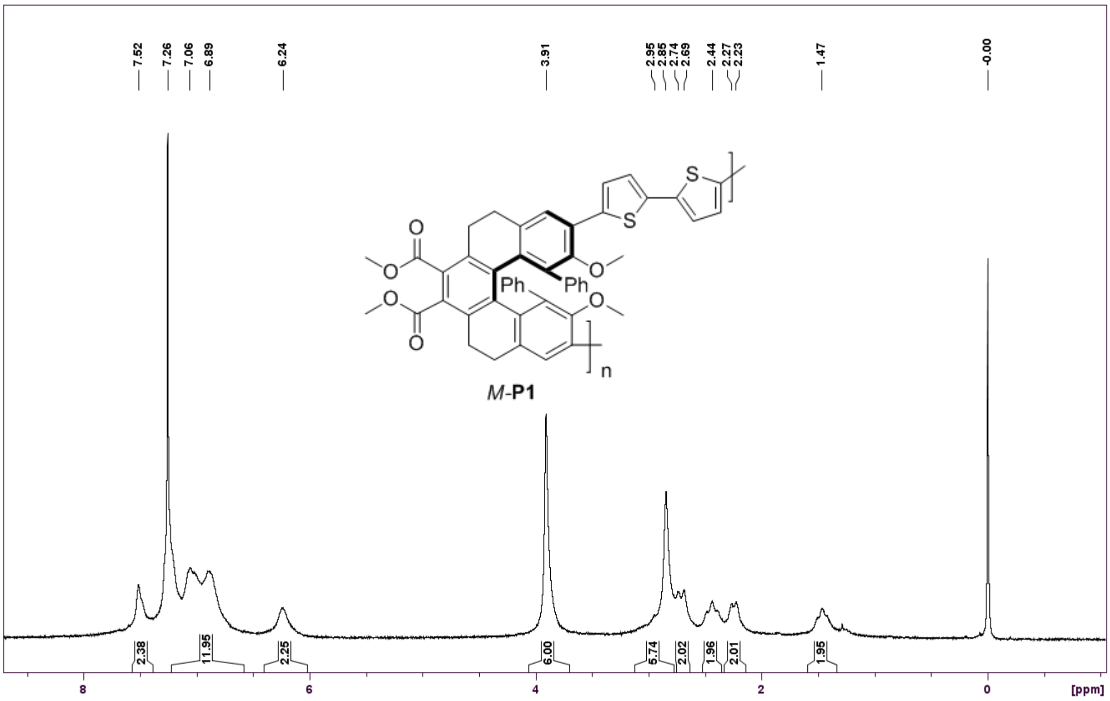

Fig. S3 ^{1}H NMR spectrum (300 MHz, $CDCl_3$) of (-)-*M*-**P1**.

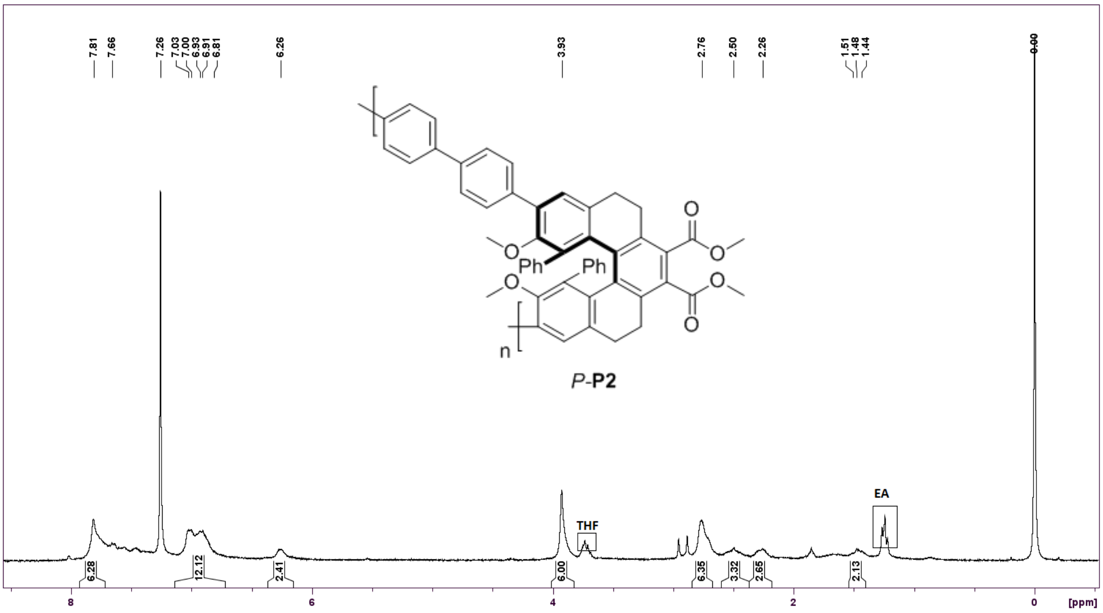

Fig. S4 ^{1}H NMR spectrum (300 MHz, $CDCl_3$) of (+)-*P*-**P2**.

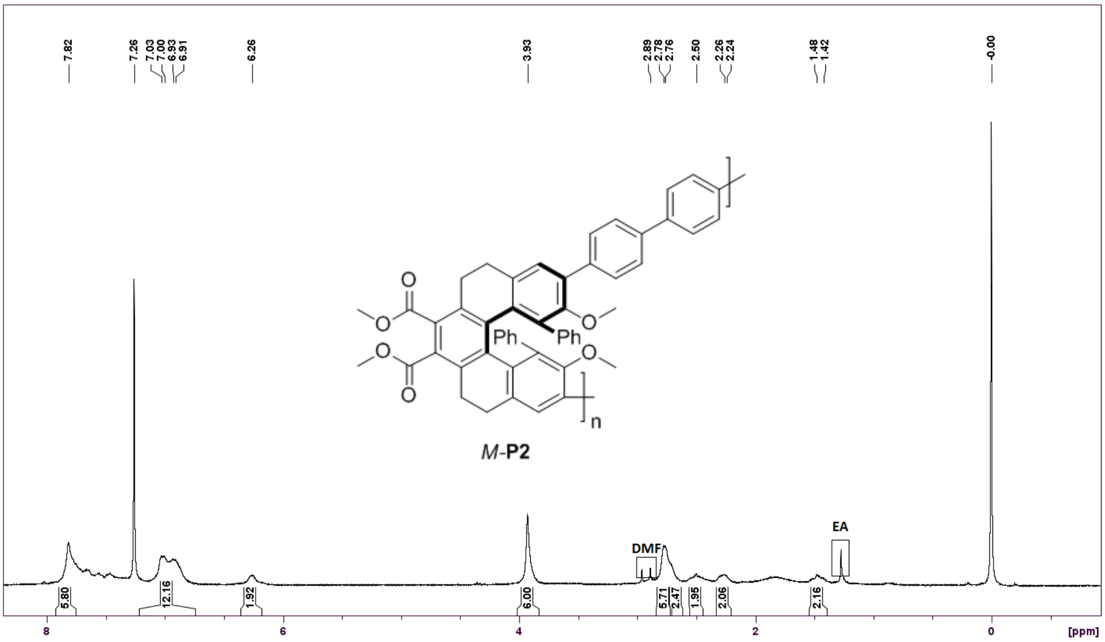

Fig. S5 ^{1}H NMR spectrum (300 MHz, $CDCl_3$) of (-)-*M*-**P2**.

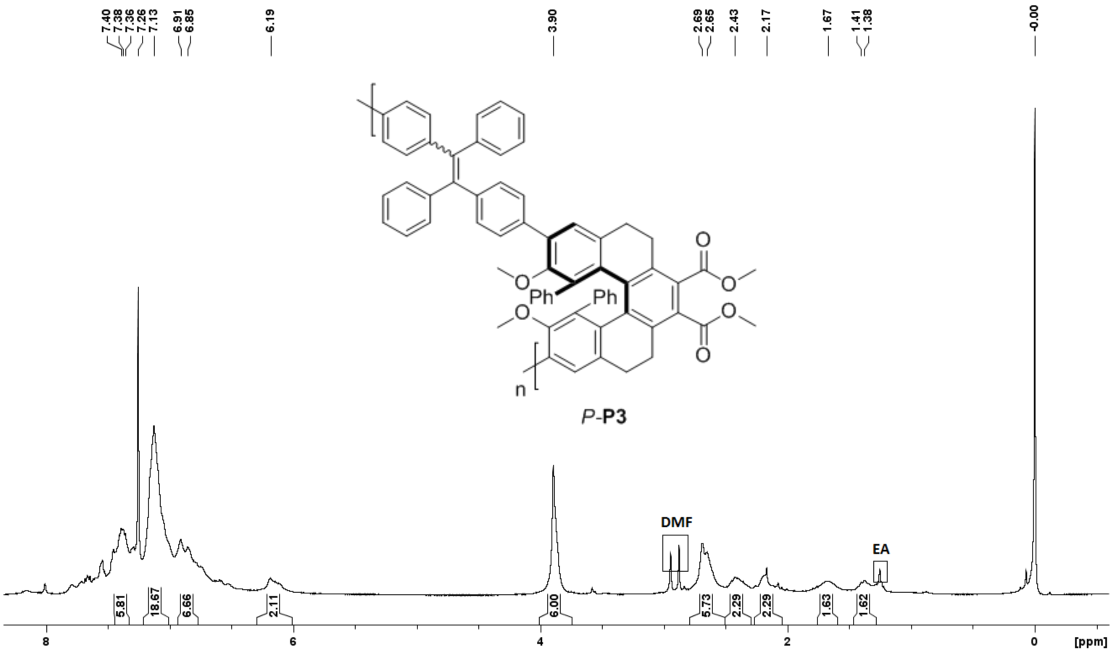

Fig. S6 ^{1}H NMR spectrum (500 MHz, $CDCl_3$) of (+)-*P*-**P3**.

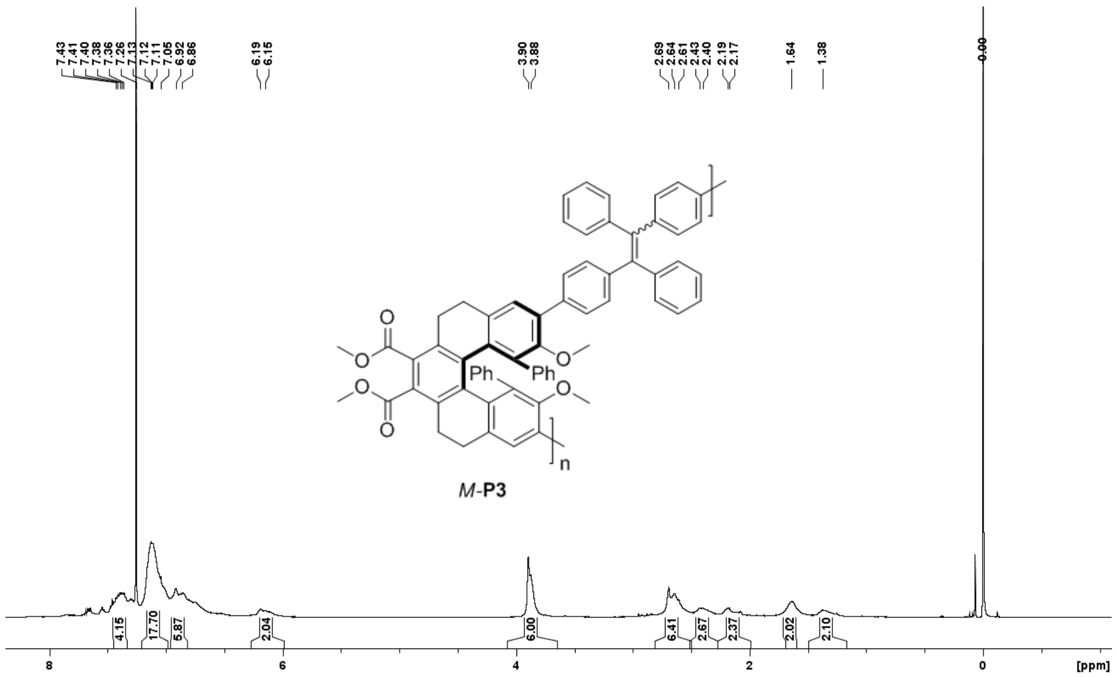

Fig. S7 ^{1}H NMR spectrum (500 MHz, $CDCl_3$) of (-)-*M*-**P3**.

S2. Copies of the FTIR spectra

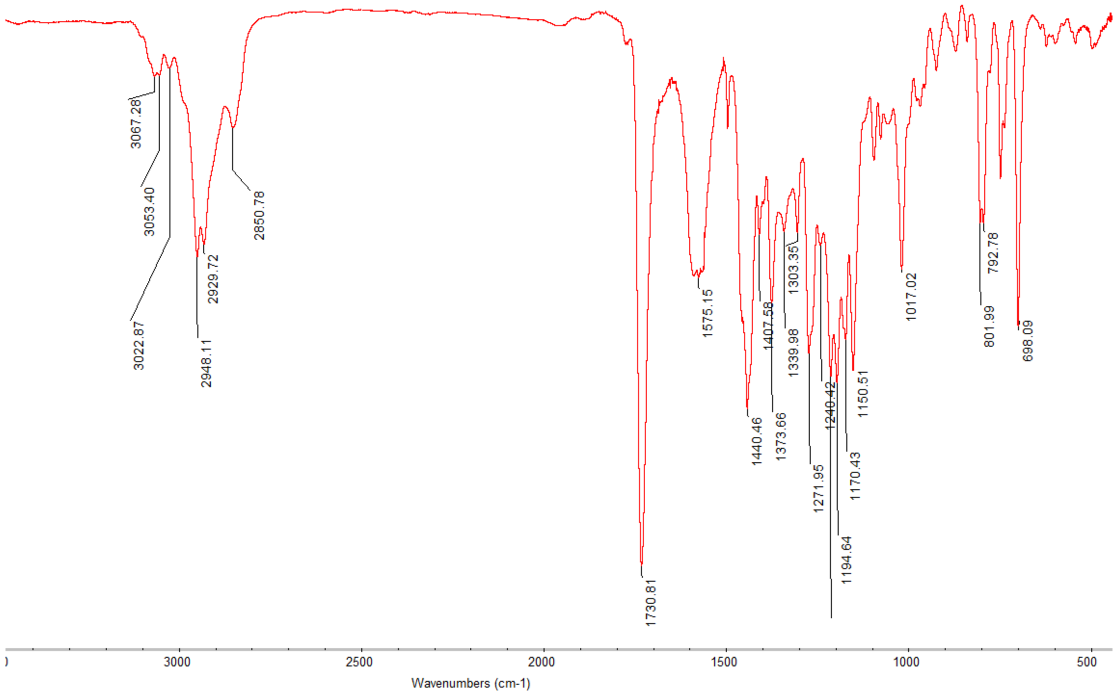

Fig. S8 FTIR spectrum of (+)-*P*-**P1**.

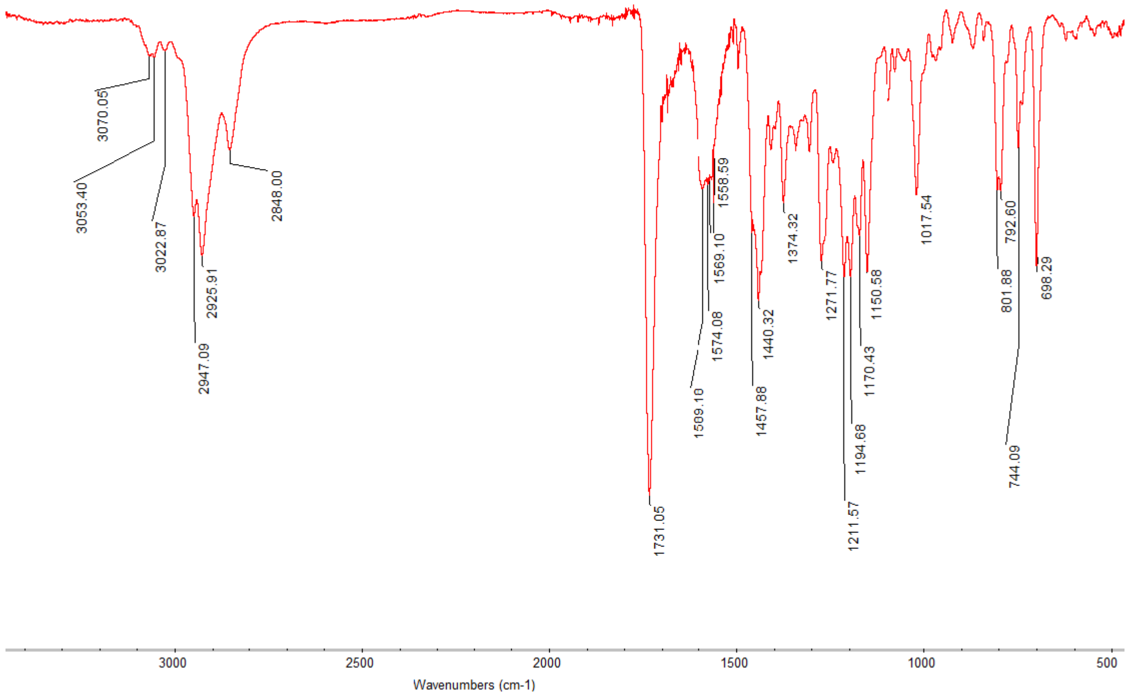

Fig. S9 FTIR spectrum of (-)-*M*-**P1**.

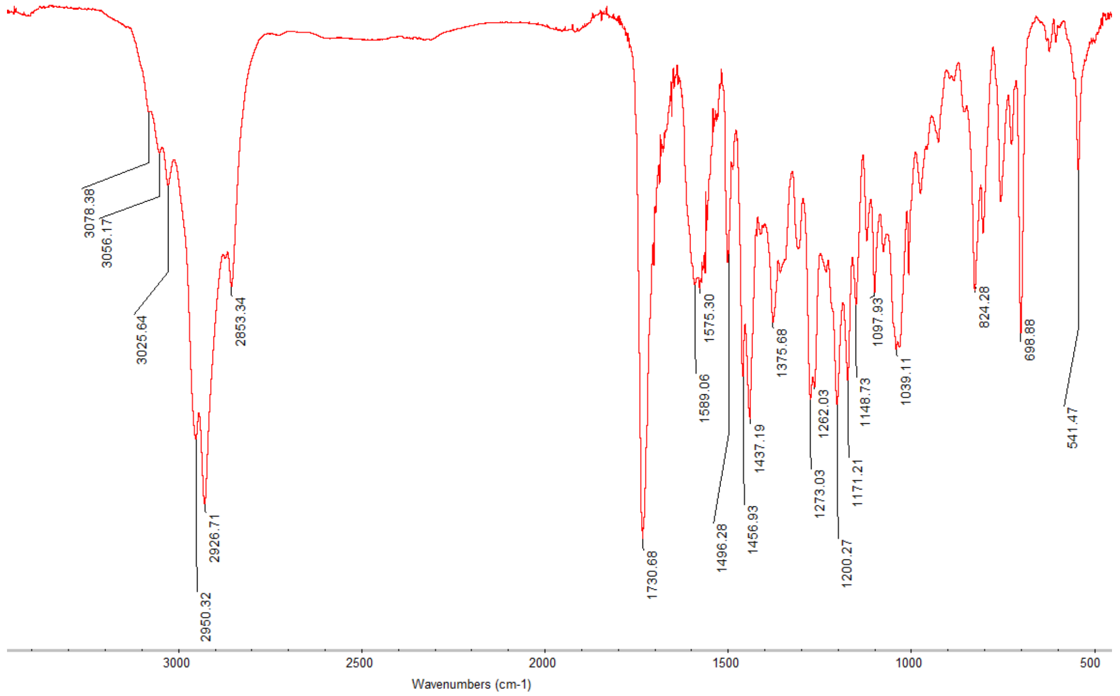

Fig. S10 FTIR spectrum of (+)-*P*-**P2**.

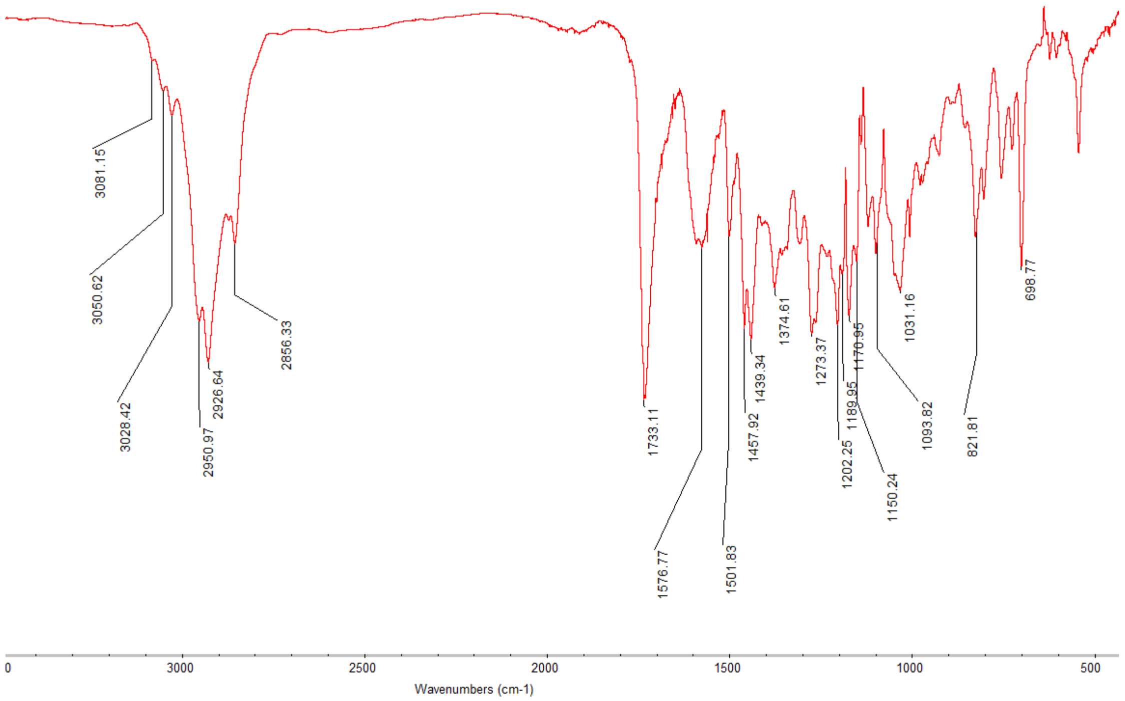

Fig. S11 FTIR spectrum of (-)-*M*-**P2**.

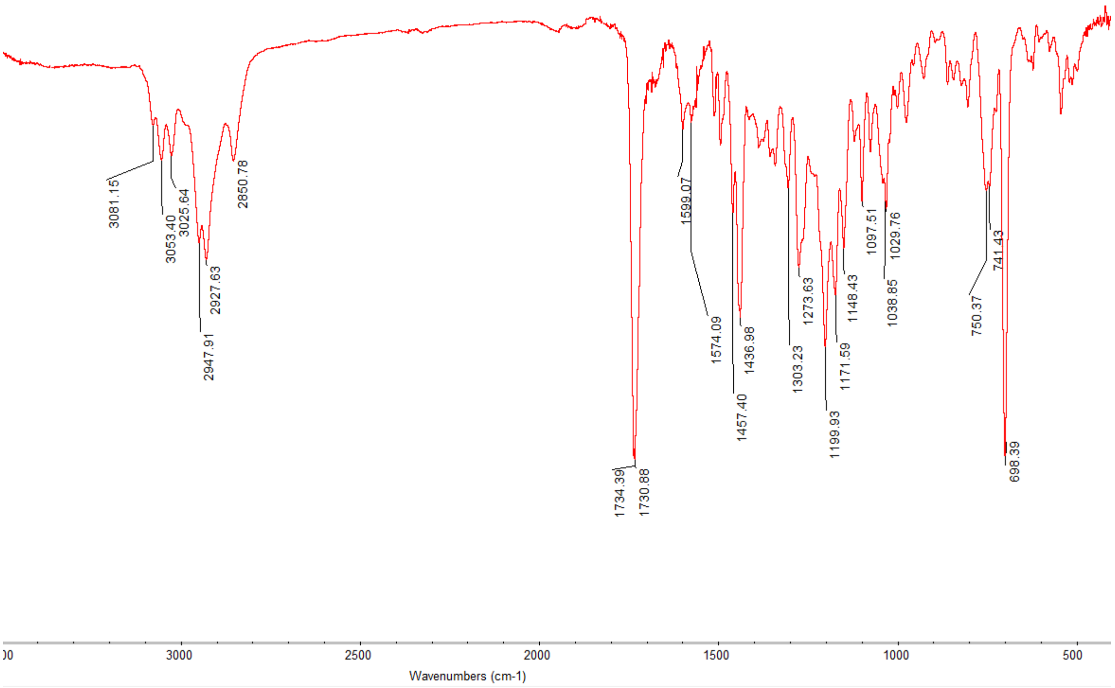

Fig. S12 FTIR spectrum of (+)-*P*-**P3.**

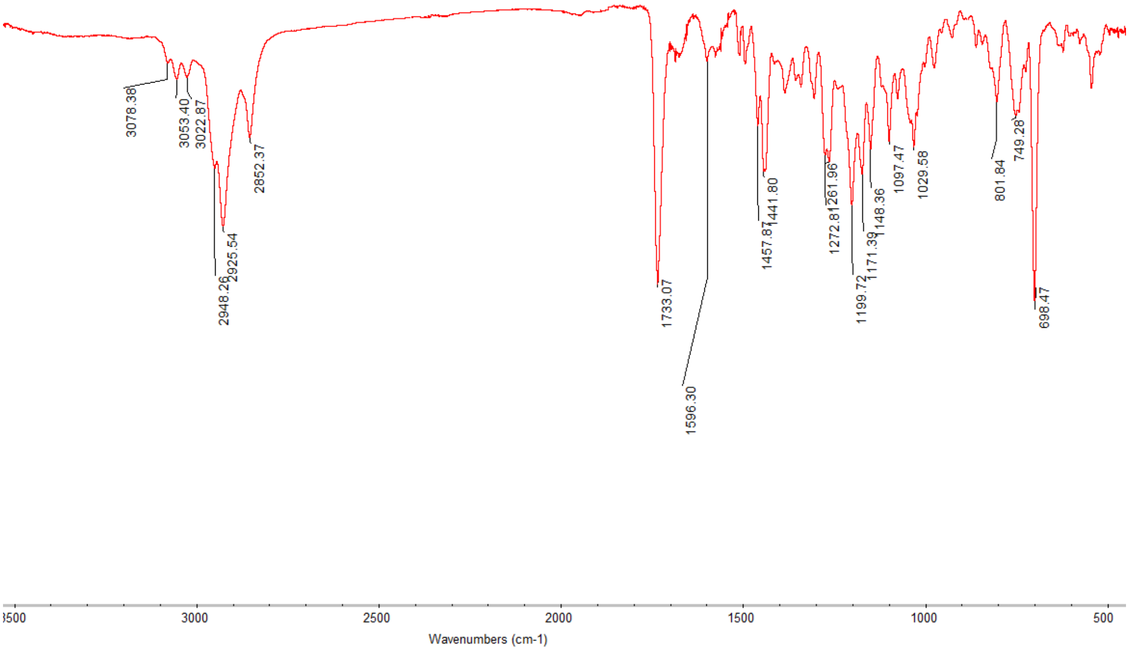

Fig. S13 FTIR spectrum of (-)-*M*-**P3.**

S3. TGA thermogram of the polymers

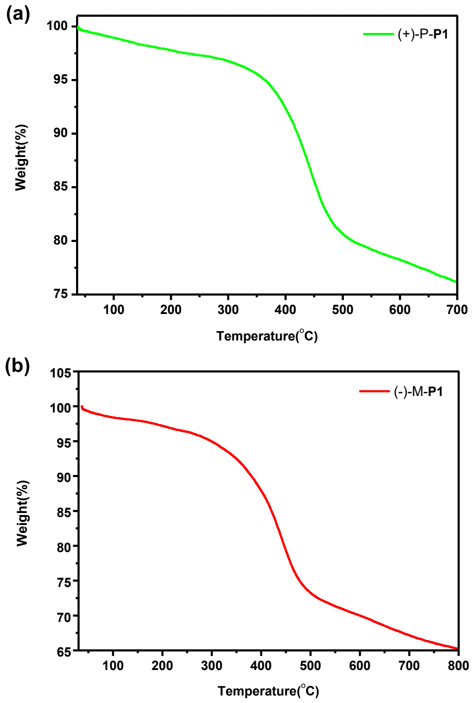

Fig. S14 (a) TGA plots of (+)-*P*-**P1** with a heating rate of 10 °C/min under nitrogen; (b) TGA plots of (-)-*M*-**P1** with a heating rate of 10 °C/min under nitrogen.

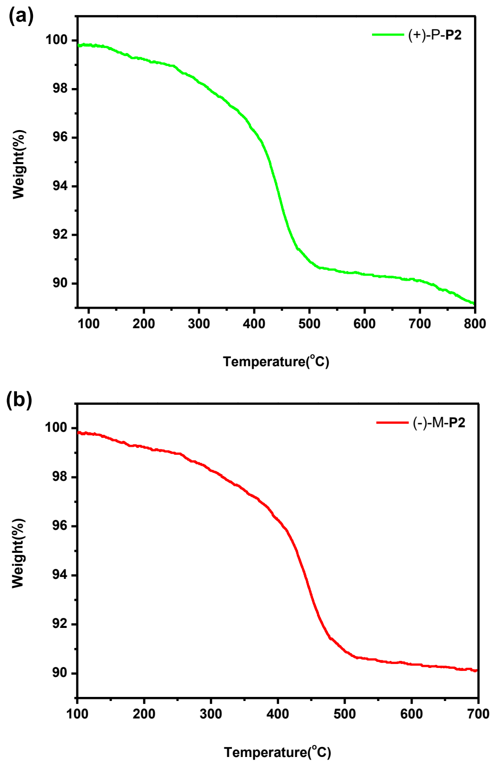

Fig. S15 (a) TGA plots of (+)-*P*-**P2** with a heating rate of 10 °C/min under nitrogen; (b) TGA plots of (-)-*M*-**P2** with a heating rate of 10 °C/min under nitrogen.

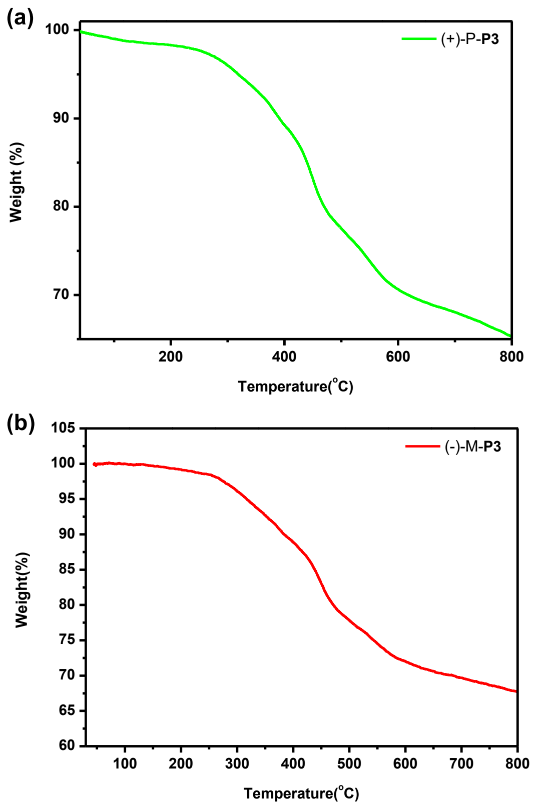

Fig. S16 (a) TGA plots of (+)-*P*-**P3** with a heating rate of 10 °C/min under nitrogen; (b) TGA plots of (-)-*M*-**P3** with a heating rate of 10 °C/min under nitrogen.

S4. UV-vis and fluorescence spectra of the polymers

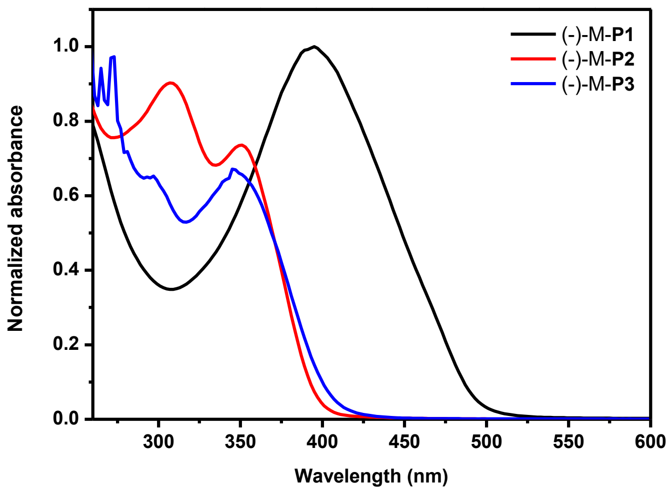

Fig. S17 UV-vis spectra of (-)-*M*-**P1-3** in THF (c = 1.0 × 10^{-4} M).

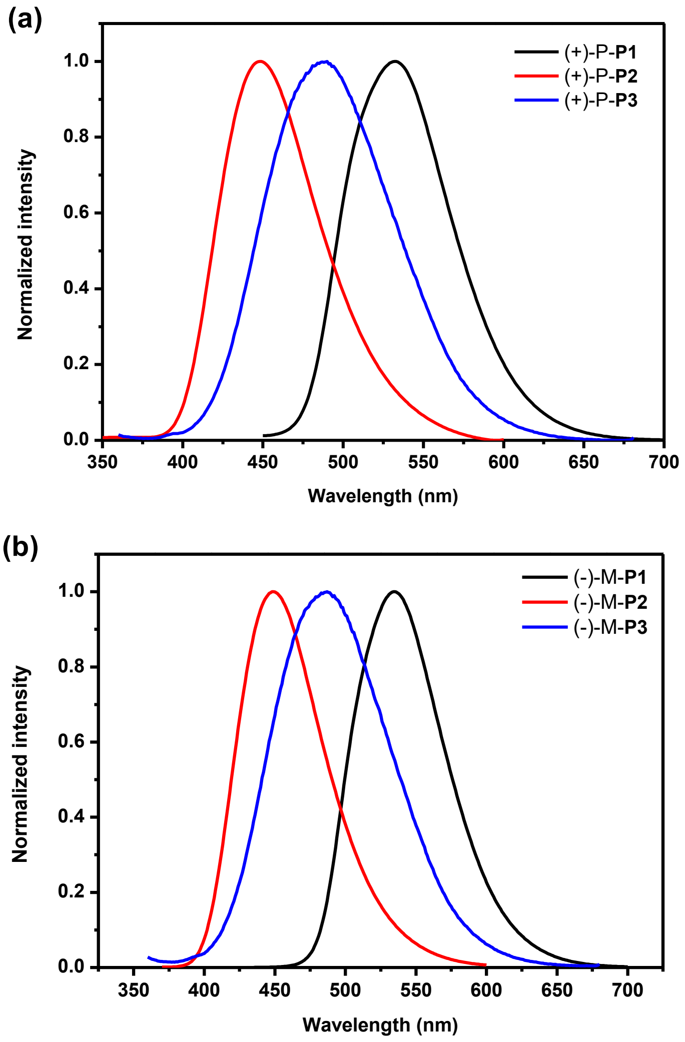

Fig. S18 (a) Fluorescence spectra of (+)-*P*-**P1-3** in THF (c = 1.0 × 10^{-4} M); (b) Fluorescence spectra of (-)-*M*-**P1-3** in THF (c = 1.0 × 10^{-4} M).

(a)

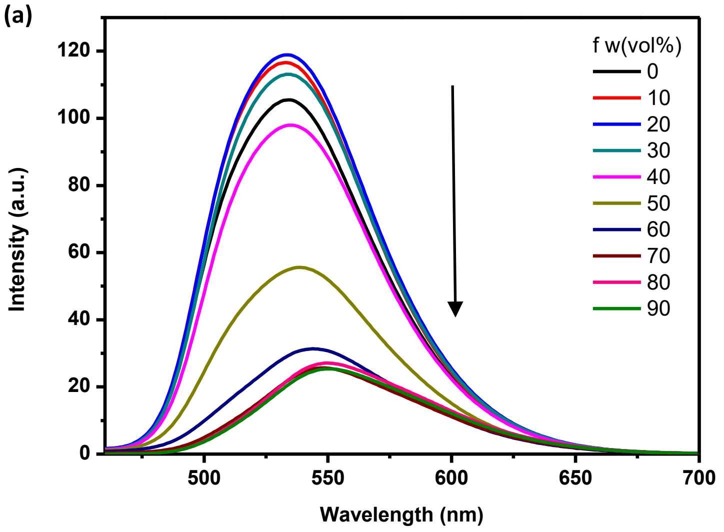

(b)

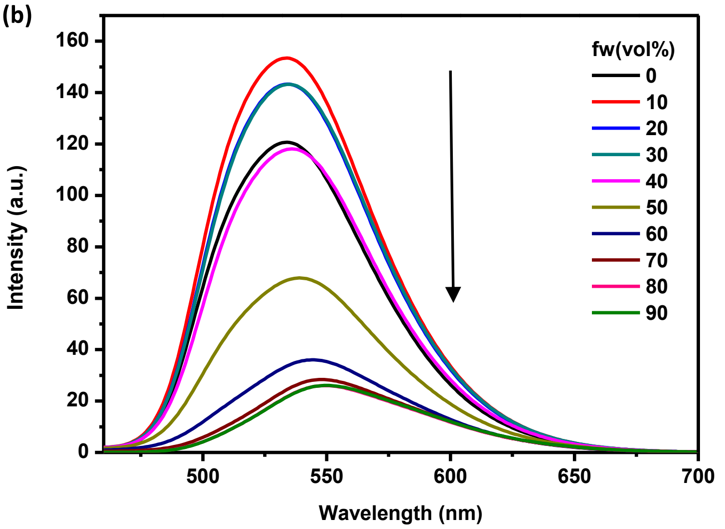

Fig. S19 (a) Fluorescence spectra of (+)-*P*-**P1** in THF-water mixtures (c = 1.0 × 10^{-4} M); (b) Fluorescence spectra of (-)-*M*-**P1** in THF-water mixtures (c = 1.0 × 10^{-4} M).

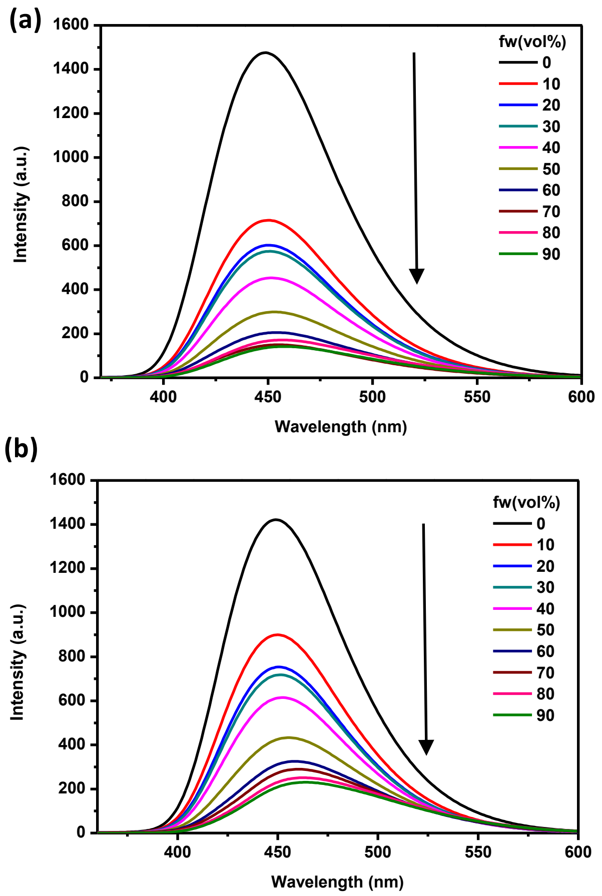

Fig. S20 (a) Fluorescence spectra of (+)-*P*-**P2** in THF-water mixtures (c = 1.0 × 10^{-4} M); (b) Fluorescence spectra of (-)-*M*-**P2** in THF-water mixtures (c = 1.0 × 10^{-4} M).

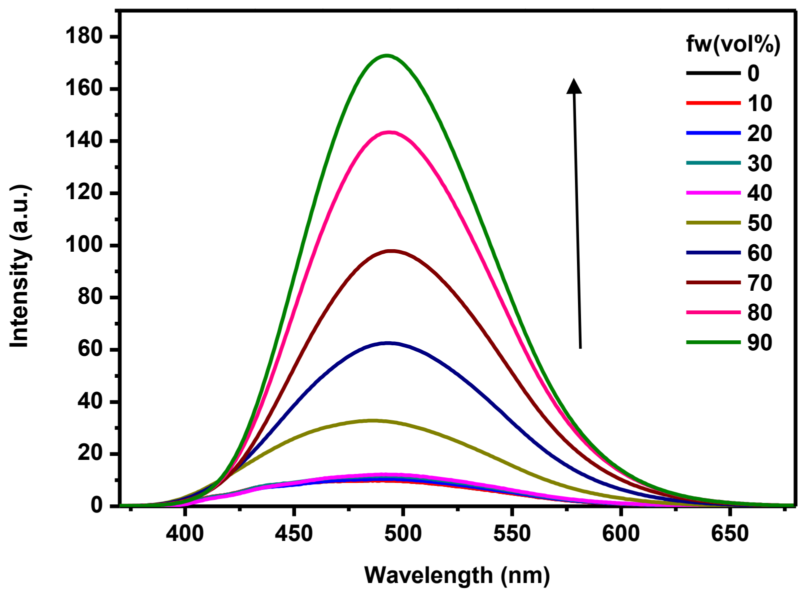

Fig. S21 Fluorescence spectra of (-)-*M*-**P3** in THF-water mixtures (c: 1.0 × 10^{-4} M corresponding to the TPE moiety).

S5. Emission color coordinate of (+)-*P*-**P2** and (-)-*M*-**P2**

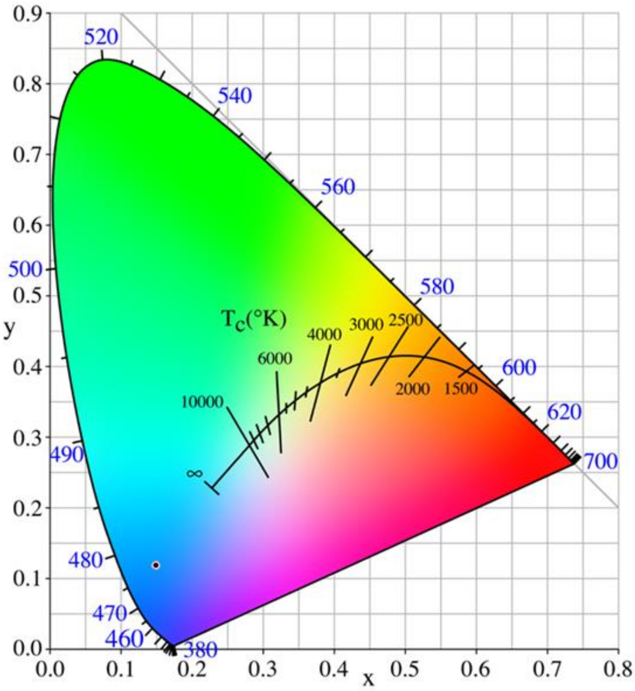

Fig. S22 Emission color coordinate of (+)-*P*-**P2** and (-)-*M*-**P2** in THF solution in the CIE 1931 chromaticity diagram.

S6. Optical rotation values of (+)-*P*-**P1-3** and (-)-*M*-**P1-3**

Table S1. Experimental optical rotation values (1 mg/mL, DMSO, 25 °C)

enantiomer	$[\alpha]_D$	enantiomer	$[\alpha]_D$
(+)-*P*-**P1**	523	(-)-*M*-**P1**	-534
(+)-*P*-**P2**	205	(-)-*M*-**P2**	-195
(+)-*P*-**P3**	405	(-)-*M*-**P3**	-390

S7. UV-vis spectra and fluorescence spectra of the nanoparticles assembled by (-)-*M*-P3

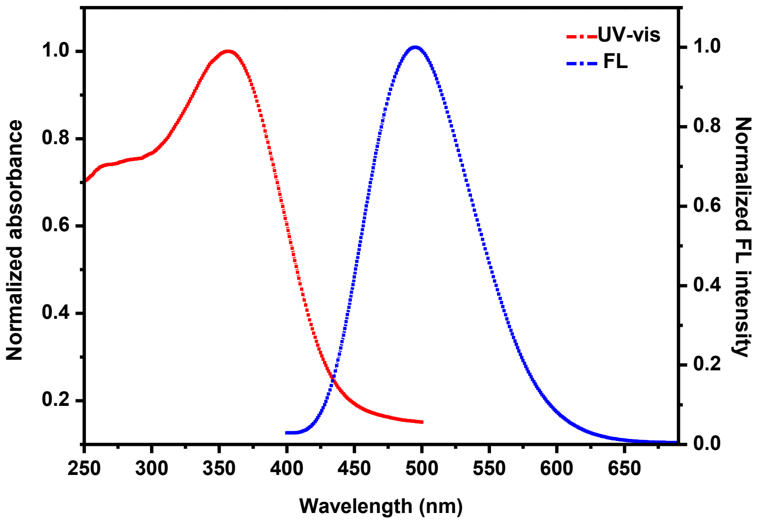

Fig. S23 UV-vis spectra and fluorescence spectra of the nanoparticles assembled by (-)-*M*-**P3**.

S8. CPL spectra of (+)-*P*-P3 and (-)-*M*-P3 in the film state

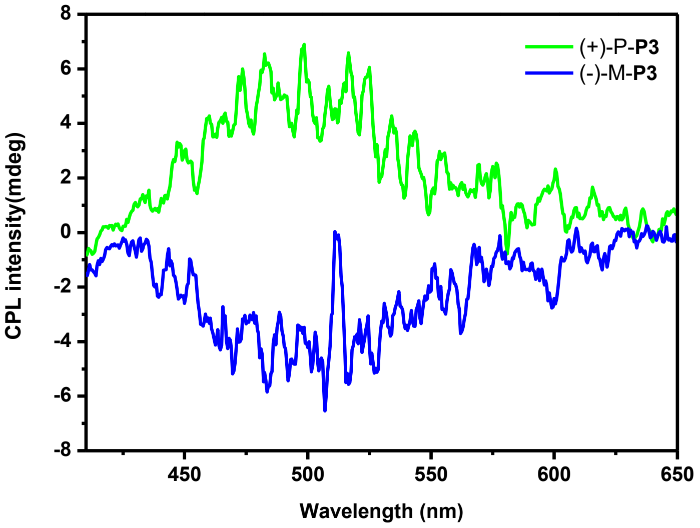

Fig. S24 CPL spectra of (+)-*P*-**P3** and (-)-*M*-**P3** in the film state.
